# ^131^I age-dependent inhalation dose in Southern Poland from Fukushima accident

**DOI:** 10.1007/s00411-016-0672-3

**Published:** 2016-12-23

**Authors:** K. Brudecki, K. Szufa, J. W. Mietelski

**Affiliations:** Institute of Nuclear Physics, Polish Academy of Sciences, Radzikowskiego 152, 31-342 Kraków, Poland

**Keywords:** ^131^I, Fukushima, Inhalation dose

## Abstract

A general method for calculating doses absorbed from isotopes released in nuclear accidents is presented. As an example, this method was used to calculate doses for inhabitants of Southern Poland due to inhalation of ^131^I released due to the Fukushima nuclear plant accident. ^131^I activity measurements in the air of that region provided the basis for the study. The proposed model is based on a complex biokinetic model for iodine merging the Leggett model developed in 2010 with the human respiratory tract and gastrointestinal tract models recommended by the International Commission on Radiological Protection (ICRP). This model is described here, and it is demonstrated that resulting dose estimates are consistent with those obtained using the ICRP methodology. Using the developed model, total doses were calculated for six age groups of both genders, for gaseous and aerosol fractions alike. The committed effective dose, *H*_50_, for an adult man reached 16 nSv, which is lower than 0.001% of the background dose. The dose for the thyroid of an adult reached 0.33 μSv, which corresponds to circa 0.0007% of the dose to the population of Southern Poland after the Chernobyl nuclear plant accident.

## Introduction

On 11 March 2011, a 9.0 magnitude earthquake took place off the Pacific coast of Tōhoku, Japan. Subsequent 13-m-high tsunami waves damaged the cooling system (Tanaka 2012; Lipscy et al. 2013) at the Fukushima Daiichi Nuclear Power Plant. As a consequence, a radioactive emission occurred on the 12th of March containing mainly ^131^I, ^133^I, ^134^Cs, ^137^Cs, and other volatile species, which were transported in the atmosphere towards the north-western direction (Tanaka 2012). The accident was classified in the International Nuclear Event Scale as a level 7 accident. Previously, only the Chernobyl accident was rated at this level. During this accident, 1.8 × 10^18^ Bq of ^131^I were released into the environment (UNSCEAR 2000), whereas during the Fukushima accident 0.12 × 10^18^ Bq of ^131^I were released (USCEAR 2014).

The first traces from the Fukushima radioactive plume in Europe were detected in Reykjavik, Iceland, on the 20th of March (Bossew et al. 2012). On the morning of the 24th of March, the Laboratory of Radioactivity Analyses in Krakow, Poland, initiated an emergency mode for observations of radionuclides in the atmosphere; both aerosols and a gaseous fraction of ^131^I were considered (Mietelski et al. 2014).

^131^I is a beta-emitting radioisotope with a physical half-life of 8.021 days. The mean energy of electrons released during ^131^I nuclear decay is 606 keV. ^131^I is a nuclear fission product and as such it is commonly present in radioactive releases due to nuclear power plant failures. Furthermore, it is believed to contribute significantly to health hazards related to radioactive fallout. Being an iodine radioisotope, ^131^I is easily and preferentially concentrated in the human thyroid; note that the thyroid accumulates 30% of the total intake of iodine (Johansson et al. 2003) which is necessary for the proper functioning of the thyroid.

The International Commission on Radiological Protection (ICRP) developed a general methodology to calculate dose conversion factors and dose coefficients. This methodology was applied in the present study to estimate doses received by an average Polish citizen due to inhalation of ^131^I from the Fukushima fallout. More specifically, to quantify the incorporation of aerosol and gaseous fractions of radioiodine in the human organism, the age-dependent systemic biokinetic model proposed by Leggett (2010) was combined with the ICRP human respiratory tract model (HRTM) (ICRP 1994) and the ICRP gastrointestinal tract model (GITM) (ICRP 1979).

## Materials and method

A general method of dose calculation is presented in this section. First, ^131^I concentration in the air was measured. Second, gaseous and aerosol ^131^I fractions deposited in various lung regions were estimated. Next, a complete biokinetic model for modelling the translocation of the deposited radionuclide within a human body following inhalation was constructed. Finally, ICRP recommendations were applied to evaluate equivalent and effective doses.

### ^131^I measurements

Iodine from the air was collected at the Institute of Nuclear Physics in Krakow, Poland, by a combined aerosol sampler MASS-500/gas sampler, operated at a flow rate of 250 m^3^ h^−1^ (Mietelski et al. 2014; Masson et al. 2011). The sorbent type for the aerosol fraction was a Petryanov filter FPP-15-1.5 (poly(vinyl chloride)) which has good aerosol-collecting properties: The aerosol collection efficiency of the Petryanov filter for aerosols with diameters 0.3 µm and for a linear velocity of the air passing through the filter of about 0.36 m s^−1^ reached 96.7% (Lipiński et al. 2013). For collection of the gas fraction, a sorbent of granular activated carbon impregnated with KI (IBJ-6, mesh size 2 mm, produced by Gryskand, Hajnówka, Poland) was used. The average efficiency of gaseous iodine adsorption reached (67 ± 11)% (Table 1). The use of activated carbon impregnated with KI allowed collection of both organic and inorganic iodine (Wilhelm 1982; Wangchang et al. 1993).

**Table 1:** Results of gamma spectrometric measurements of ground air activity concentration in Kraków, Poland, for one-day sampling cycle (Masson et al. 2011, 2013; Mietelski et al. 2014)

Start	Stop	*V* (m^3^)	^131^I activity concentration, aerosol fraction (µBq m^−3^)	^131^I aerosol AMAD diameter (μm)	^131^I activity concentration, gas fraction (µBq m^−3^)	Total efficiency of gaseous iodine adsorption (%)
2011.03.21	2011.03.24	17,957	105 ± 5	0.38	135 ± 42	69
2011.03.24	2011.03.25	5975	84 ± 7		286 ± 103	41
2011.03.25	2011.03.26	6386	<12		<48	46
2011.03.26	2011.03.27	5256	840 ± 37		604 ± 170	75
2011.03.27	2011.03.28	7468	1570 ± 60		2040 ± 860	61
2011.03.28	2011.03.29	5385	3600 ± 200	0.36	5200 ± 1200	69
2011.03.29	2011.03.30	5763	5730 ± 350		5220 ± 340	68
2011.03.30	2011.03.31	7264	2910 ± 120		3360 ± 260	78
2011.03.31	2011.04.01	6177	908 ± 42	0.35	1120 ± 110	73
2011.04.01	2011.04.02	6396	668 ± 33		227 ± 20	69
2011.04.02	2011.04.03	6628	718 ± 35		145 ± 15	74
2011.04.03	2011.04.04	6230	2151 ± 90		406 ± 27	69
2011.04.04	2011.04.05	8201	413 ± 18	0.47	95 ± 15	81
2011.04.05	2011.04.06	4261	494 ± 27		224 ± 44	76
2011.04.06	2011.04.07	6689	650 ± 27		250 ± 27	71
2011.04.07	2011.04.08	6554	438 ± 48		316 ± 46	61
2011.04.08	2011.04.09	6067	308 ± 20		90 ± 12	70
2011.04.09	2011.04.10	6788	182 ± 16	0.53	191 ± 20	72
2011.04.10	2011.04.11	6625	239 ± 15		358 ± 34	45
2011.04.11	2011.04.13	12,882	157 ± 13		59 ± 15	60

Between the 21st of March and 12th of April, the sorbents were changed every day. The filters and the granulated carbon from the gas cassettes were analysed by means of a low-background gamma spectrometer with HPGe detectors. Prior to measurements, the filters were compressed into pellets of 5 cm diameter and about 4 mm height. The carbon from each cassette was transferred into 0.5-L plastic Marinelli beakers and measured. Isotopes such as ^131^I, ^132^I, ^129m^Te, ^132^Te, ^134^Cs, ^136^Cs, and ^137^Cs were detected in the filters. The maximum activity for aerosols was equal to (5.73 ± 0.35), (0.461 ± 0.041) and (0.436 ± 0.038) mBq m^−3^ for ^131^I, ^134^Cs, and ^137^Cs, respectively, as observed on 29 March 2011. The detailed results are presented in Table 1 (Masson et al. 2011, 2013; Mietelski et al. 2014).

### Deposition in the human respiratory tract

In the present study, the HRTM published in ICRP (1994) was used. In this approach, the human respiratory tract is divided into four anatomical regions, namely the extrathoracic region ET (consists of two parts: ET1 comprising interior nose and ET2 comprising posterior nasal passages, larynx, pharynx, and mouth), the bronchial region BB, the bronchiolar region bb, and the alveolar–interstitial region AI. The deposition of ^131^I aerosol fractions was calculated for five activity median aerodynamic diameters (AMADs) of the attached aerosols that were observed in Southern Poland after the Fukushima accident, i.e. 0.35, 0.36, 0.38, 0.47, and 0.53 μm (Masson et al. 2013). The deposition was calculated by a logarithmic interpolation of the deposition fraction values published by the ICRP in the HRTM using a six-term exponential function. Exemplary depositions for an adult male and a 0.35 μm aerosol AMAD are presented in Table 2.

**Table 2:** Depositions in the ICRP human respiratory tract model (HRTM) for an adult male and a 0.35 μm AMAD aerosol diameter

Region	Exercise levels			
	Sleeping	Sitting	Light exercise	Heavy exercise
ET1	3.41 × 10^−2^	3.02 × 10^−2^	7.23 × 10^−2^	3.36 × 10^−2^
ET2	3.12 × 10^−2^	3.91 × 10^−2^	8.21 × 10^−2^	4.69 × 10^−2^
BB fast and seq	4.56 × 10^−3^	4.03 × 10^−3^	2.69 × 10^−3^	5.78 × 10^−3^
BB slow	4.04 × 10^−3^	3.77 × 10^−3^	3.11 × 10^−3^	6.84 × 10^−3^
bb fast and seq	1.65 × 10^−2^	2.45 × 10^−2^	1.24 × 10^−2^	1.02 × 10^−2^
bb slow	2.77 × 10^−2^	2.52 × 10^−2^	1.14 × 10^−2^	1.08 × 10^−2^
AI	1.33 × 10^−1^	1.53 × 10^−1^	1.29 × 10^−1^	1.42 × 10^−1^

ET1, extrathoracic region; ET2, posterior nasal passages; BB, bronchial; bb, bronchiolar; AI, alveolar–interstitial

In the gas fraction, iodine in elemental and organic form is absorbed in the respiratory tract in various proportions. However, when it is not possible to distinguish them (as was the case in the present study), the ICRP recommends adoption of the deposition rate for elemental iodine; accordingly, 100% deposition in the respiratory tract for elemental iodine was assumed in the present study with the respective distribution of 10% ET1, 40% ET2, and 50% BB (ICRP 1995). This simplification resulted in an overestimation of the calculated doses. In general, the fractional deposition in the respiratory tract regions was estimated for six age groups, both genders and four exercise levels, notably sitting, sleeping, light exercise, and heavy exercise. The typical time-budget distribution of different activities for 3-month infants was assumed as 71–29% for sleeping and light exercises, respectively, while the typical time budget for an adult male was assumed to be 33, 25, 41, and 1% for sleeping, sitting, light exercise, and heavy exercise, respectively. The average breathing rate for an adult man is 22.2 m^3^ day^−1^, while the breathing rates for the other age groups are 2.86 m^3^ day^−1^ for 3-month-old infants, 5.16 m^3^ day^−1^ for 1-year-olds, 8.72 m^3^ day^−1^ for 5-year-olds, 15.3 m^3^ day^−1^ for 10-year-olds, 20.1 m^3^ day^−1^ for 15-year-old males, 18 m^3^ day^−1^ for 15-year-old females, and 17.8 m^3^ day^−1^ for adult females. These breathing rates and other detailed ventilation parameters used in the model were taken from ICRP (2002) and are presented in Table 3.

**Table 3:** Daily time budget and ventilation parameters at each exercise level for members of the public (ICRP 2002)

Age	Time budget (h)				Breathing rate (m^3^ h^−1^)				
	Sleeping	Sitting	Light exercise	Heavy exercise	Sleeping	Sitting	Light exercise	Heavy exercise	Average
3 months	17	–	7	–	0.09	–	0.19	–	0.12
1 year	14	3.33	6.67	–	0.15	0.22	0.35	–	0.22
5 years	12	4	8	–	0.24	0.32	0.57	–	0.36
10 year	10	4.67	9.33	–	0.31	0.38	1.1	–	0.64
15 years male	10	5.5	7.5	1	0.42	0.48	1.4	2.9	0.84
15 years female	10	5.5	7.5	1	0.35	0.40	1.3	2.6	0.75
Adult male	8	6	9.75	0.25	0.45	0.54	1.5	3.0	0.93
Adult female	8	6	9.75	0.25	0.32	0.39	1.2	2.7	0.74

### Biokinetic models

**Fig. 1:**
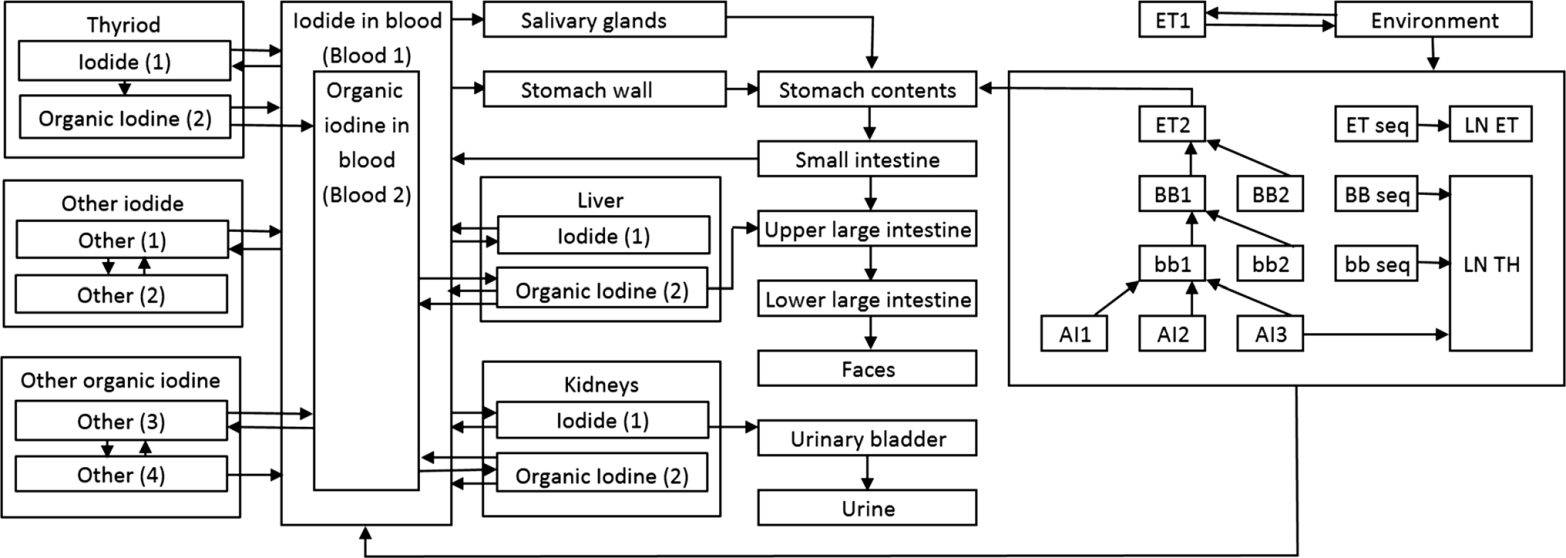
General biokinetic model used in the present study. The model combines ICRP human respiratory track model and gastrointestinal tract model and Leggett systemic model (Leggett 2010; ICRP 1979, 1994)

To describe the behaviour of the inhaled ^131^I in the human body and iodine accumulation in organs and tissues, the systemic biokinetic compartment model by Leggett (2010), the HRTM, and GITM were combined together into a one-compartment model. Such a solution has already been successfully applied for calculation of doses due to inhalation of indoor short-lived radon (both ^222^Rn and ^220^Rn) progenies for members of the public (Lie et al. 2008; Brudecki et al. 2014). The model is based on three subsystems for extrathyroidal inorganic iodine, thyroidal iodine, and extrathyroidal organic iodine. The model flow chart is presented in Fig. 1.

In the model, transfer of organic and hormonal iodine between compartments is characterized by transfer coefficients defined as the transferred fraction of the source compartment content per unit time. The factors used here are presented in Table 4. It is presupposed that inhaled iodine is quickly and entirely absorbed into the blood, where the iodine in red blood cells (RBCs) and plasma is assumed to be the same; therefore, the blood iodine is treated as a mixed pool. Another assumption is that iodine is excreted solely through urine and faeces (more than 90% of iodine removal is caused by renal clearance). Sweat secretion seems to be a negligible contribution to iodine loss. It is worth pointing out that only liver and kidney compartments were divided into two regions representing organic and inorganic iodide. The reasons for such specification are that kidneys are a greater extrathyroidal repository of organic and inorganic iodine, whereas liver stores much more hormonal iodine than any other extrathyroidal organ.

**Table 4:** Parameter values of the Leggett ^131^I systemic model (Leggett 2010)

Pathway	Transfer coefficient (day^−1^)
Blood 1 to thyroid 1	7.26
Blood 1 to urinary bladder contents	11.84
Blood 1 to salivary glands	5.16
Blood 1 to stomach wall	8.60
Blood 1 to other 1	600
Blood 1 to kidneys 1	25
Blood 1 to liver 1	15
Salivary glands to stomach contents	50
Stomach wall to stomach contents	50
Thyroid 1 to thyroid 2	95
Thyroid 1 to blood 1	36
Thyroid 2 to blood 2	0.0077
Thyroid 2 to blood 1	0
Other 1 to blood 1	330
Other 1 to other 2	35
Other 2 to other 1	56
Kidney 1 to blood 1	100
Liver 1 to blood 1	100
Blood 2 to other 3	15
Other 3 to blood 2	21
Other 3 to other 4	1.2
Other 4 to other 3	0.62
Other 4 to blood 1	0.14
Blood 2 to kidneys 2	3.6
Kidneys 2 to blood 2	21
Kidneys 2 to blood 1	0.14
Blood 2 to liver 2	21
Liver 2 to blood 2	21
Liver 2 to blood 1	0.14
Stomach contents to SI contents	20.57
SI contents to blood 1	594
Urinary bladder contents to urine	12

The model proposed by Leggett in 2010 is a biokinetic model for iodine for adults. Note that differences in the biokinetics of iodine between age groups are caused primarily by differences in iodine retention in the thyroid. The biological half-life of iodine for the thyroid is 11.2, 15, 23, 58, 67, and 90 days, for infants, 1-, 5-, 10-, and 15-year-old children and adults, respectively (ICRP 1993; Leggett 2010). In Leggett’s model, the time of iodine retention in the thyroid corresponds to the “Thyroid 2 to 2 Blood” parameter. Based on the values for biological half-life of iodine given above, this factor corresponds to 0.0619, 0.0462, 0.0301, 0.0119, 0.0103, and 0.0077 day^−1^ for infants, 1-, 5-, 10-, and 15-year-old children and adults, respectively.

The biokinetic behaviour of the inhaled iodine in the respiratory tract and other organs can be described by a system of first-order linear differential equations. The iodine activity in tissue and organs at any time after intake can be calculated numerically by solving these equations. The time-integrated iodine activity $\tilde{A}$ in the thyroid and in other organs was calculated hereby integrating the activity in the organ up to the age of 70 years for children and over the 50-year commitment period following the intake for adults. The system of first-order differential equations was solved using the commercially available software SAAM II (Epsilon Group, VA, USA) (Barrett et al. 1998).

### Equivalent dose and effective dose

The general methodology for dose calculation developed by the ICRP was also applied in the present study. Equivalent thyroid doses were calculated based on the time-integrated activity $\tilde{A}$ and radiation-weighted *S*_W_ factors. In general, the equivalent dose *H*(*r*_*T*_, *T*_*D*_) in any target organ *r*_*T*_ at age *T* integrated over the dose integration period *T*_*D*_ is given by Eq. (1) (Bolch et al. 2009)

1 $$H\left( {r_{T} ,T_{D} } \right) = \sum\limits_{{r_{S} }} {\int\limits_{0}^{{T_{D} }} {A\left( {r_{S} ,t} \right)S_{\text{W}} \left( {r_{T} \leftarrow r_{S} ,t} \right){\text{d}}t} } ,$$

where *A*(*r*_*S*_, *t*) is the time-dependent activity in the source region *r*_*S*_ at time *t*; *S*_W_(*r*_*T*_ ← *r*_*S*_, *t*) is the radiation-weighted *S* factor, which depends on the radiation-weighting factor *w*_*R*_ and energy *E*_*R*_ of radiation *R. S* is calculated as given in Eq. (2)

2 $$S_{\text{W}} \left( {r_{T} \leftarrow r_{S} ,t} \right) = \sum\limits_{R} {w_{R} S\left( {r_{T} \leftarrow r_{S} ,E_{R} ,t} \right)} .$$

For a given age group, *S*_W_(*r*_*T*_ ← *r*_*S*_, *t*) is constant over the time interval *T*_*D*_^age^ and $\tilde{A}$ may be calculated as (Eq. 3)

3 $$\tilde{A}\left( {r_{S} ,T_{D}^{\text{age}} } \right) = \int\limits_{0}^{{T_{D}^{\text{age}} }} {A\left( {r_{S} ,t} \right){\text{d}}t} .$$

Consistently, the equivalent dose in a specific age group is (Eq. 4)

4 $$H\left( {r_{T} ,T_{D}^{\text{age}} } \right) = \sum\limits_{{r_{S} }} {\tilde{A}\left( {r_{S} ,T_{D}^{\text{age}} } \right)S_{W} \left( {r_{T} \leftarrow r_{S} } \right)} .$$

Finally, the equivalent dose is calculated according to Eq. (5) by summing all the equivalent doses of each time interval as

5 $$H\left( {r_{T} ,T_{D} } \right) = \sum\limits_{{{\text{all}}\;{\text{ages}}}} {H\left( {r_{T} ,T_{D}^{\text{age}} } \right)} .$$

Subsequently, the effective dose for a certain gender, *E*, may be calculated by Eq. (6) by summing equivalent doses, *H*_*T*_, for every target region and the tissue-weighting factor, *w*_*T*_

6 $$E = \sum\limits_{T} {w_{T} H_{T} } .$$

Following the new ICRP recommendations (ICRP 2007), the effective dose, *E*, for adults is calculated from the average of adult female ($H_{T}^{\text{Female}}$) and adult male ($H_{T}^{\text{Male}}$) equivalent doses (Eq. 7)

7 $$E = \sum\limits_{T} {w_{T} \left( {\frac{{H_{T}^{\text{Female}} + H_{T}^{\text{Male}} }}{2}} \right)}$$

In the present investigation, the effective doses for adults and 15-year-olds are estimated based on the adequate female and male equivalent doses and ICRP 103 (2007) recommendations. For the other age groups, i.e. infants, 1-, 5-, and 10-year-olds, the effective doses are calculated without taking into consideration gender differences, according to ICRP (1991).

In practice, the *S*_W_ value was calculated using the SEECAL program (Oak Ridge National Laboratory, Oak Ridge, TN, USA). The time-dependent activity, *A*(*r*_*S*_, *t*), was obtained by modelling for separate anatomical regions (Fig. 1) and the *w*_*T*_ values used for calculations were taken from ICRP 60 (1991). Tissue-weighting factors, *w*_*T*_, were taken from ICRP 60 (1991) instead of ICRP 103 (2007), to allow validation of the model and comparison of the results with ICRP dose coefficients (ICRP 1995).

### Validation of the method

To validate the methods applied, the estimated doses were compared with those obtained using ICRP dose coefficients (ICRP 1995) for the gaseous fraction of ^131^I. Results are presented in Table 5. The biggest differences are observed for infants (36%) and 1-year-old children (16%). In the other age groups, differences are around 10%. Such discrepancies occurred owing to the fact that Leggett’s model is more accurate than the current ICRP model proposed by Riggs (1952).

**Table 5:** Effective dose in Sv for the ^131^I gas fraction

	3 month	1 year	5 years	10 years	15 years ♂	15 years ♀	Adult ♂	Adult ♀
Eff dose (present method)	1.9 × 10^−8^	2.8 × 10^−8^	2.6 × 10^−8^	2.3 × 10^−8^	1.9 × 10^−8^	1.7 × 10^−8^	1.4 × 10^−8^	1.1 × 10^−8^
Eff dose (ICRP 71, dose coefficients)	1.4 × 10^−8^	2.4 × 10^−8^	2.3 × 10^−8^	2.1 × 10^−8^	1.8 × 10^−8^	1.6 × 10^−8^	1.3 × 10^−8^	1.0 × 10^−8^
Difference	36%	16%	13%	10%	6%	6%	8%	10%

## Results and discussion

In the present study, doses to South Poland residents due to inhalation of ^131^I were calculated, based on the ^131^I measured in air in South Poland after the nuclear power plant accident in Fukushima. The resulting effective doses and equivalent doses for the thyroid obtained for six age groups, namely 3-month infants, 1-, 5-, 10-, 15-year-old, and adults, as well as both genders for adults and 15-year-old teenagers, are shown in Tables 6 and 7.

**Table 6:** Inhalation dose in Sv for the ^131^I aerosol fraction

	3 month	1 year	5 years	10 years	15 years ♂	15 years ♀	Adult ♂	Adult ♀
Thyroid	1.0 × 10^−7^	1.9 × 10^−7^	1.5 × 10^−7^	1.0 × 10^−7^	7.7 × 10^−8^	6.9 × 10^−8^	5.7 × 10^−8^	4.6 × 10^−8^
Effective dose	5.3 × 10^−9^	9.6 × 10^−9^	7.3 × 10^−9^	5.2 × 10^−9^	3.9 × 10^−9^	3.5 × 10^−9^	2.8 × 10^−9^	2.3 × 10^−9^

**Table 7:** Inhalation dose in Sv for the ^131^I gas fraction

	3 month	1 year	5 years	10 years	15 years ♂	15 years ♀	Adult ♂	Adult ♀
Thyroid	3.8 × 10^−7^	5.5 × 10^−7^	5.1 × 10^−7^	4.5 × 10^−7^	3.9 × 10^−7^	3.4 × 10^−7^	2.7 × 10^−7^	2.1 × 10^−7^
Effective dose	1.9 × 10^−8^	2.8 × 10^−8^	2.6 × 10^−8^	2.3 × 10^−8^	1.9 × 10^−8^	1.7 × 10^−8^	1.4 × 10^−8^	1.1 × 10^−8^

**Fig. 2:**
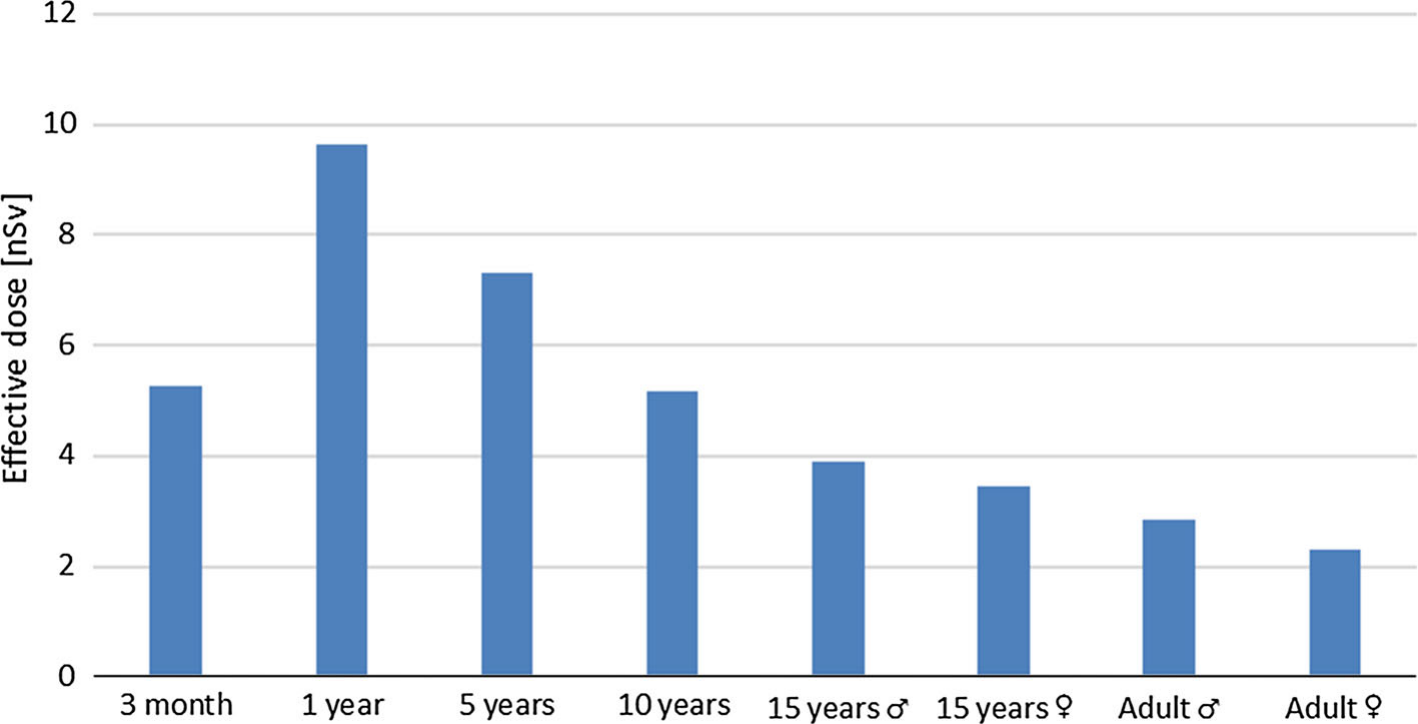
Effective dose due to aerosol fraction of ^131^I as a function of age

With regard to the aerosol fraction, as expected the highest equivalent dose was achieved for the thyroid. The estimated doses for this organ were equal to 1.0 × 10^−7^, 1.9 × 10^−7^, 1.5 × 10^−7^, 1.0 × 10^−7^, 7.7 × 10^−8^, 6.9 × 10^−8^, 5.7 × 10^−8^, and 4.6 × 10^−8^ Sv for infants, 1-, 5-, 10-year-old children, 15-year-old males, 15-year-old females, male and female adults, respectively. The equivalent doses received by other body parts usually did not exceed hundreds of pSv. The largest effective dose (9.6 nSv) was calculated for a 1-year-old child, while for older ages, lower values were calculated (Fig. 2).

**Fig. 3:**
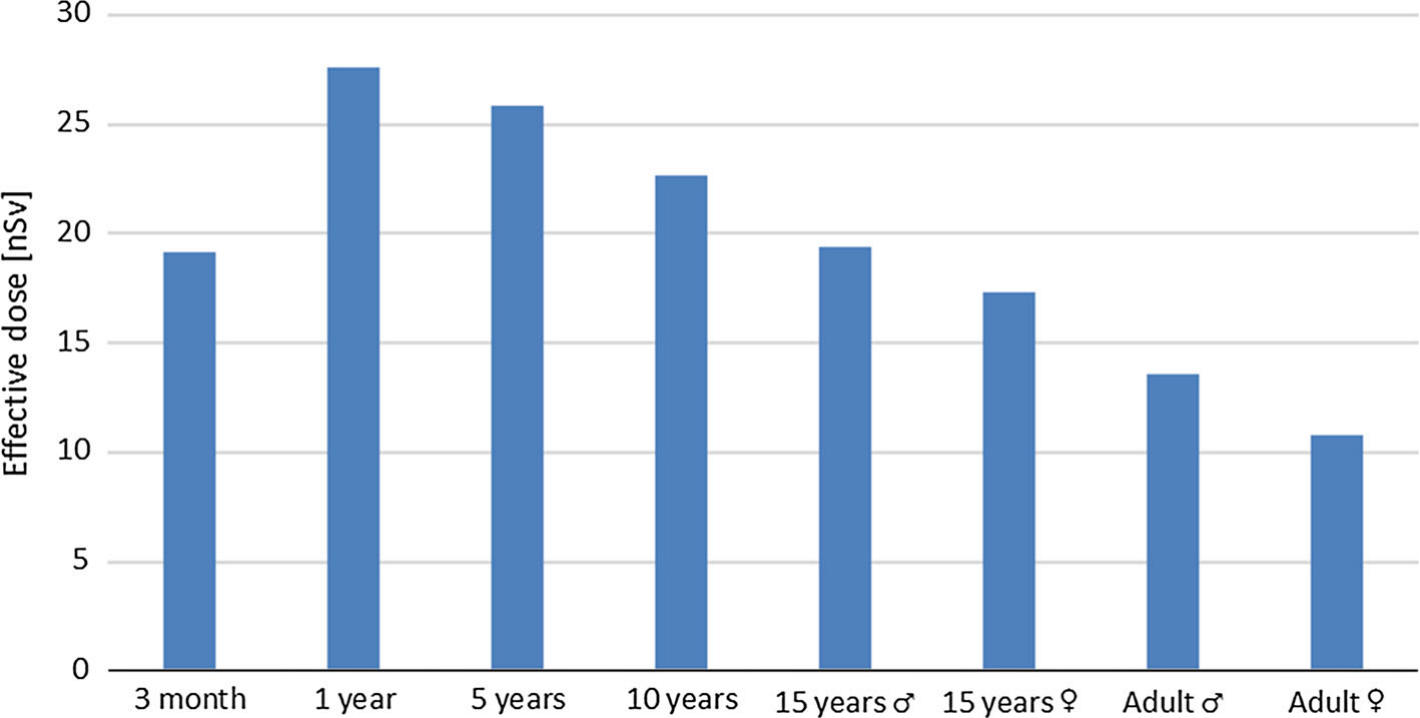
Effective dose due to gas fraction of ^131^I as a function of age

As far as the gaseous fraction of ^131^I is concerned, the thyroid was the organ with the highest equivalent doses, similarly to the case of the aerosol fraction. The thyroid equivalent doses were equal to 3.8 × 10^−7^, 5.5 × 10^−7^, 5.1 × 10^−7^, 4.5 × 10^−7^, 3.9 × 10^−7^, 3.4 × 10^−7^, 2.7 × 10^−7^, and 2.1 × 10^−7^ Sv for infants, 1-, 5-, 10-year-old children, 15-year-old males, 15-year-old females, male and female adults, respectively. Corresponding effective doses ranged from 28 nSv for a 1-year-old child to 11 nSv for an adult female (Fig. 3).

It should be noted that the doses calculated due to the gaseous fraction are overestimated. As already mentioned above, elemental and organic iodine in the gas fraction are absorbed in the respiratory tract in various proportions: 100% of elemental iodine is absorbed while only 70% of organic iodine is absorbed. For an adult male, the difference between the effective dose due to inhalation of elemental iodine and organic iodine is 25% (ICRP 1995). Because we were not able to distinguish between elemental and organic iodine in the gas fraction, it was assumed that all was elemental iodine and a 100% absorption was used, consistent with the recommendations of the ICRP. This led to a conservative dose assessment. Considering that organic iodine constitutes up to 86% of the total gaseous fraction (Noguchi and Murata 1988), doses estimated in the present study were probably overestimated by up to about 23%.

In general, the effective doses from iodine in the gaseous fraction are higher than those from iodine attached to aerosols: The gas-to-aerosol effective dose ratios are equal to 3.6, 2.9, 3.5, 4.4, 5.0, 4.8, and 4.7 for infant, 1-, 5-, 10-year-old children, 15-year-old boys and girls, adult men and women, respectively. Such differences in dose between the gaseous and aerosol fractions are caused by differences in deposition in the respiratory system. Accumulation of volatile (gaseous) iodine is between 70% and 100%, while the maximum deposition of particulate iodine (iodine attached to aerosols) observed in Southern Poland is on the 43% level for 0.53-μm-diameter particles for a 1-year-old child.

The sum of the thyroid doses induced by the iodine attached to aerosols or present in the gaseous fraction (and assuming 100% absorption) is equal to 0.48, 0.74, 0.66, 0.55, 0.46, 0.41, 0.33, and 0.26 μSv for infants, 1-, 5-, 10-year-old children, 15-year-old boys and girls, adult men and women, respectively. Compared with inhalation doses of ^131^I by Polish residents after the Chernobyl nuclear accident, when equivalent doses for the thyroid reached 178, 120, and 45 mSv for 5-year-old children, 10-year-old children, and adults, respectively (Pietrzak-Flis et al. 2003), the values due to the Fukushima accident are practically negligible. Similarly, the effective doses estimated here for Southern Poland are significantly lower than those estimated for the inhabitants of Fukushima and Japan: Depending on the residence place, thyroid doses for Japanese people were estimated as (0.1 ÷ 4.3), (0.1 ÷ 5.9), and (0.2 ÷ 7.5) mSv for adults, 10-year-old children, and 1-year-old children, respectively (UNSCEAR 2014). The doses found in the present study are consistent with those estimated by WHO (2012), who estimated for areas outside Japan effective doses of less than 0.01 mSv.

In the present paper, dose evaluation was performed for adults and 15-year-old teenagers for male and female separately, although ICRP recommends (ICRP 103) to calculate doses for adults as the average of the two genders (see Eq. 7). Differences found between these two approaches are presented in Table 8; the biggest discrepancy reached 13%.

**Table 8:** Comparison of effective doses between the sex-dependent calculation

	15 years ♂	15 years ♀	Average	Difference (%)	Adult ♂	Adult ♀	Average	Difference (%)
Effective dose from intake of aerosols fraction (Sv)	3.9 × 10^−9^	3.5 × 10^−9^	3.7 × 10^−9^	5	2.8 × 10^−9^	2.3 × 10^−9^	2.55 × 10^−9^	10
Effective dose from intake of gas fraction (Sv)	1.9 × 10^−8^	1.7 × 10^−8^	1.8 × 10^−8^	6	1.4 × 10^−8^	1.1 × 10^−8^	1.25 × 10^−8^	13

## Conclusions

In the present study, doses due to ^131^I originating from the Fukushima Daiichi Nuclear Power Plant accident inhaled by residents of Southern Poland were estimated. The doses were calculated for both ^131^I in aerosol and ^131^I in gaseous fractions. The dose values due to volatile iodine in the gaseous fraction were found to be higher than those estimated for particulate iodine (i.e. iodine attached to aerosols); the difference can be attributed to differences in the deposition of the discussed fractions in the human respiratory tract. The effective committed dose, *H*_50_, for a male adult reached 16 nSv, which is less than 0.001% of the local background dose. The thyroid dose for an adult reached 0.33 μSv, which is less than 0.0007% of the dose that had been estimated for residents of Southern Poland, due to the Chernobyl accident. The doses to the inhabitants of Southern Poland did by no means represent any health hazard. Moreover, ^131^I air concentrations were comparable in most European countries (especially Central and Eastern parts of Europe). Therefore, the presented dose calculations are representative not only for Poles but also for most Europeans.
